# General health and social outcomes 50 years after exposure to antenatal betamethasone: follow-up of a randomised controlled trial

**DOI:** 10.1186/s12916-024-03732-1

**Published:** 2024-11-04

**Authors:** Anthony G. B. Walters, Greg D. Gamble, Caroline A. Crowther, Stuart R. Dalziel, Carl L. Eagleton, Christopher J. D. McKinlay, Barry J. Milne, Jane E. Harding

**Affiliations:** 1 Liggins Institute, University of Auckland, Auckland, New Zealand; 2 Department of Paediatrics: Child and Youth Health, University of Auckland, Auckland, New Zealand; 3 Department of Surgery, University of Auckland, Auckland, New Zealand; 4 Centre of Methods and Policy Application in Social Sciences, University of Auckland, Auckland, New Zealand; 5 Children’s Emergency Department, Starship Children’s Hospital, Auckland, New Zealand

**Keywords:** Antenatal corticosteroids, Betamethasone, Preterm birth, Long-term outcomes, Follow-up studies, Neonatal respiratory distress syndrome

## Abstract

**Background:**

Antenatal corticosteroids are recommended for women at risk of preterm birth from 24 to 34 weeks’ gestation as they reduce neonatal morbidity and mortality, but evidence regarding their long-term effects on offspring is limited. This study assessed general health and social outcomes 50 years after antenatal exposure to corticosteroids.

**Methods:**

We assessed 424 adult offspring of women who participated in the first randomised, double-blind, placebo-controlled trial of antenatal betamethasone for the prevention of neonatal respiratory distress syndrome. The first 717 mothers received two intramuscular injections of betamethasone (6 mg betamethasone sodium phosphate and 6 mg betamethasone acetate) or placebo given 24 h apart and the subsequent 398 received two injections of double dose betamethasone (12 mg betamethasone sodium phosphate and 12 mg betamethasone acetate) or equivalent volume of placebo. Follow-up included a health questionnaire and consent for access to administrative data sources. Outcome categories included mental health (depression, anxiety, bipolar affective disorder, schizophrenia and treatment or hospital admission for any mental health disorder), general health (chronic kidney disease, cancer diagnosis, bone fracture, oral health, allergies, functional difficulties and physical activity) and social outcomes (educational attainment, employment and criminal convictions). Investigators remained blinded to treatment allocation. Analyses were adjusted for gestational age at entry, sex and clustering.

**Results:**

We assessed 424 adult offspring (46% of survivors; mean [SD] age 49.3 [1.0] years; 212 [50%] female). There was no difference in mental health, general health and social outcomes between those exposed to betamethasone and those exposed to placebo, with the exception that osteoporotic site fracture in adulthood was more likely to have occurred in the betamethasone group compared with placebo (adjusted relative risk 1.57, 95% CI 1.00, 2.48, *p* = 0.05). No dose–effect relationship was evident and there was no difference in the proportion with at least one fracture. Follow-up rate and lack of in-person assessments were the main limitations.

**Conclusions:**

There is no evidence that antenatal corticosteroids have clinically important effects on general health and social outcomes up to 50 years of age.

## Background

Preterm birth affects more than one in ten births globally and is associated with significant neonatal mortality and morbidity, including respiratory distress syndrome, intraventricular haemorrhage and necrotising enterocolitis [1, 2]. Antenatal corticosteroids (ANC) given to women at risk of preterm birth reduce neonatal mortality and morbidity [3]. As a result, ANCs are recommended for women at risk of preterm birth between 24 and 34 weeks’ gestation [4]. Given the significant global incidence of preterm birth and the consequently large number of infants exposed to ANC, understanding their effects on long-term health is critical. This is particularly important given the increasing understanding of the impacts of early-life events on chronic disease and the potential role of glucocorticoid exposure in the pathophysiology of this relationship [5]. Many animal and human studies have assessed cardiovascular, respiratory and metabolic outcomes in adulthood after exposure to ANC [6–13]. However, evidence for many other outcomes of importance to long-term health and wellbeing is sparse, with much of the evidence being observational and reporting outcomes only in childhood [3, 14].

The Auckland Steroid Trial (AST, December 1969–February 1974) was the first randomised, placebo-controlled trial of ANC for women at risk of preterm birth [15, 16]. The corticosteroid used in the AST, and in many subsequent trials of ANC, was a combination of short-acting betamethasone sodium phosphate (6 mg) and long-acting betamethasone acetate (6 mg). Given the potential for early life exposures to influence a multitude of outcomes, many of which are more common at older ages, we assessed the impact of antenatal betamethasone exposure on a range of health and social outcomes in the offspring of the AST at 50 years. Cardiovascular and respiratory outcomes at 50 years are reported elsewhere [17].

## Methods

This study is reported as per the Strengthening the Reporting of Observational Studies in Epidemiology guideline (Additional file 1).

### The Auckland Steroid Trial

The AST and earlier follow-up have been described elsewhere [15, 16]. Briefly, between December 1969 and February 1974, a double-blind, randomised, placebo-controlled trial of antenatal corticosteroids (6 mg betamethasone sodium phosphate and 6 mg betamethasone acetate by intramuscular injection, repeated after 24 h) for women at risk of preterm birth was performed at National Women’s Hospital, Auckland, New Zealand. In October 1972, after 717 women (769 infants) had been enrolled, the dose of the betamethasone and control treatments was doubled for the remainder of the trial [15]. The trial enrolled 1115 women and 1218 fetuses.

### Fifty-year follow-up

#### Participants

All surviving offspring of women who took part in the AST were eligible to participate. Participants provided written informed consent. The approach to recruitment has been described elsewhere [17]. Study staff and investigators for the 50-year follow-up study remained blinded to intervention allocation.

#### Procedures

Participants were asked to complete a questionnaire, based on the New Zealand health survey 2019/2020 (including the International Physical Activity Questionnaire [IPAQ] and Washington Group Short Set on Functioning [WGSS]) [18–20] and to consent for the study team to access administrative datasets managed by the New Zealand Ministry of Health [National Minimum Dataset (NMDS; hospital admissions (> 3 h stay) since 1988), National Non-admitted Patients Collection (NNPaC; emergency department (< 3 h stay) and outpatient visits since 2006), Mortality dataset (MORT; national mortality records since 1970), Pharmaceutical dataset (PHARM; national records of community dispensing of pharmaceuticals funded by the New Zealand government since 1992), New Zealand Cancer Registry (NZCR; national cancer registration data since 1948)], TestSafe (laboratory tests data for Auckland and Northland regions which include 36% of New Zealand’s population and New Zealand’s largest city where all participants were born, since 2010), Accident Compensation Corporation (ACC; accident claims data since 1974), New Zealand Qualifications Authority (NZQA; national qualifications data since 1991) and Ministry of Justice (national convictions data).

#### Outcomes

Outcomes were based on self-reported questionnaires and administrative data, or both. The full list of outcomes for this study is available in Additional file 2 [21, 22]. The primary outcome and secondary outcomes related to cardiometabolic and respiratory health have been reported elsewhere [17]. Tertiary outcomes included diagnosis or treatment of a mental health disorder (self-reported, NMDS and PHARM), depression (self-report and NMDS), bipolar affective disorder (self-report and NMDS), anxiety disorder (self-report and NMDS), schizophrenia (self-report and NMDS), inpatient admission for a mental health disorder (NMDS), prescription of pharmaceuticals for mental health disorders (PHARM), fractures (total fractures in adulthood, childhood and those at osteoporotic sites as defined in Additional file 3 [19, 20, 23–25] [self-report, ACC and NMDS]), cancer diagnosis (self-report, NMDS and NZCR), diagnosis of or treatment of an allergic condition other than asthma (self-report and PHARM), chronic kidney disease (NNPaC and TestSafe), self-reported functional difficulties (WGSS), self-reported physical activity (IPAQ), self-reported oral health (general and number of teeth removed for decay), self-reported employment status, the proportion with tertiary qualifications, no secondary school qualifications (self-reported and NZQA data), and at least one criminal conviction (Ministry of Justice data). The full list of data sources for outcomes along with full outcome definitions are provided in Additional file 3.

#### Statistical analysis

The statistical analysis plan was prepared before the completion of data collection or any analyses (Additional file 2). All analyses were pre-specified unless otherwise stated and were performed using SAS version 9.4 (SAS Institute, Cary, NC) and an intention-to-treat approach with participants analysed according to the initial treatment group to which their mother was allocated. Denominators are the number of participants who consented to follow up with data available for the outcome, unless otherwise specified. No imputation was made for missing data.

Binary outcomes were compared between randomised groups using generalised linear mixed (GLM) modelling (binomial distribution, log link function) to estimate relative risk (RR) and 95% confidence intervals (CIs) both unadjusted and adjusted for gestational age at randomisation and fetal sex. Clustering of births from the same mother was adjusted for using a random effect, approximating generalised estimation equations. Continuous outcomes were compared by GLM modelling (normal distribution, identity link function) with and without the same adjustments and reported as mean difference (MD) with 95% CIs. Outcomes were tested at the 5% significance level. Significance testing was based on type 3 fixed effects obtained from the GLM model for the binary and continuous outcomes. No adjustment to the significance level was made as the study prioritised identifying important adverse effects at the risk of increased likelihood of type 1 error with multiple comparisons.

A post hoc subgroup analysis compared fracture risk in participants exposed to standard with double standard dose betamethasone and in males and females. A post hoc sensitivity analysis included trial participants deceased prior to 50-year follow-up in the analysis of cancer outcomes.

## Results

The AST randomised 1115 women and 1218 offspring, with 301 offspring dying between randomisation and 50-year follow-up. Participants (*n* = 424) in this follow-up study have previously been described [17]. In brief, participants had similar characteristics to those eligible but not participating (*n* = 493), including gestational age at trial entry (median [5th centile, 95th centile] 33.1 weeks [27.6, 36.4]), gestational age at delivery (35.3 weeks [30.0, 41.0]), birthweight (mean, SD 2406 g, 735), proportion of multiple pregnancies (110/917, 12.0%), cause of preterm labour or delivery, tocolytic use and treatment dose of betamethasone (standard or twice standard dose) (Table 1). However, the proportion of females was higher in participants than in those eligible but not participating (212/424, 50.0% vs 201/493, 40.8%). Among the participants, these characteristics were similar between those exposed in utero to betamethasone (*n* = 229) and those exposed to placebo (*n* = 195), except that there were fewer females in the betamethasone group (105/229, 45.9% vs 107/195, 54.9% in the placebo group). The mean age at follow-up was 49.3 years (SD 1.0) in both groups.

**Table 1:** Characteristics of offspring who did and did not participate in the 50-year follow-up

	**Participated**			**Eligible but did not participate** ***N* = 493**	**Deceased** ***N* = 301**	**Total in the original trial** ***N* = 1218**
	**Exposed to antenatal betamethasone** ***N* = 229**	**Exposed to placebo** ***N* = 195**	**Total** ***N* = 424**			
Stillbirth	-	-	-	-	97 (32.2%)	97 (8.0%)
Randomised to antenatal corticosteroid	229 (100%)	0 (0%)	229 (54.0%)	233 (47.3%)	139 (46.2%)	601 (49.3%)
Female	105 (45.9%)	107 (54.9%)	212 (50.0%)	201 (40.8%)	128 (42.5%)	541 (44.4%)
Gestational age at entry, weeks, median (5th, 95th centile)	32.9 (27.6, 36.0)	33.4 (27.4, 36.0)	33.1 (27.6, 36.0)	33.4 (27.5, 37.0)	29.6 (24.0, 36.0)	32.7 (26.0, 36.1)
Gestational age at delivery, weeks, median (5th, 95th centile)	34.9 (29.0, 40.6)	35.0 (30.0, 40.9)	35.0 (29.3, 40.6)	36.0 (30.7, 41.0)	31.3 (24.0, 40.0)	35.0 (27.1, 40.9)
Preterm delivery	166 (72.5%)	135 (69.2%)	301 (71.0%)	295 (59.8%)	267 (88.7%)	863 (70.9%)
Late preterm (34–37 weeks)	87 (38.0%)	83 (42.6%)	170 (40.1%)	181 (36.7%)	64 (21.3%)	415 (34.1%)
Moderate preterm (32–34 weeks)	46 (20.1%)	26 (13.3%)	72 (17.0%)	71 (14.4%)	43 (14.3%)	186 (15.3%)
Very preterm (28–32 weeks)	31 (13.5%)	24 (12.3%)	55 (13.0%)	41(8.3%)	94 (31.2%)	190 (15.6%)
Extremely preterm (< 28 weeks)	2 (0.9%)	2 (1.0%)	4 (0.9%)	2 (0.4%)	66 (21.9%)	72 (5.9%)
Multiple pregnancy						
Singleton	195 (85.2%)	175 (89.7%)	370 (87.3%)	437 (88.6%)	261 (86.7%)	1068 (87.7%)
Multiple	34 (14.9%)	20 (10.3%)	54 (12.7%)	56 (11.4%)	40 (13.3%)	150 (12.3%)
Unplanned premature labour	193 (84.3%)	161 (82.6%)	354 (83.5%)	435 (88.2%)	247 (82.1%)	1036 (85.1%)
Mode of delivery						
Normal vaginal delivery	102 (44.5%)	91 (46.7%)	193 (45.5%)	225 (45.6%)	168 (55.8%)	586 (48.1%)
Instrumental delivery	104 (45.4%)	80 (41.0%)	184 (43.4%)	210 (42.6%)	112 (37.2%)	506 (41.5%)
Caesarean section	23 (10.0%)	24 (12.3%)	47 (11.1%)	58 (11.8%)	21 (7.0%)	126 (10.3%)
Maternal intervention dose^a^						
Standard dose	145 (63.3%)	123 (63.1%)	268 (63.2%)	298 (60.5%)	203 (67.4%)	769 (63.1%)
Twice standard dose	84 (36.7%)	72 (36.9%)	156 (36.8%)	195 (39.6%)	98 (32.6%)	449 (36.9%)
Birthweight, grams, mean (SD)	2313 (776)	2322 (714)	2317 (747)	2483 (717)	1659 (833)	2221 (826)
Birthweight *Z* score, mean (SD)	− 0.32 (0.93)	− 0.42 (1.03)	− 0.36 (0.98)	− 0.42 (0.96)	− 0.31 (1.28)	− 0.37 (1.05)
5-min Apgar score < 7	31 (13.5%)	22 (11.5%)	53 (12.6%)	59 (12.1%)	92 (46.0%)	204 (16.7%)
Respiratory distress syndrome	15 (6.6%)	22 (11.3%)	37 (8.7%)	30 (6.1%)	75 (24.9%)	142 (11.7%)

^a^Standard dose = 2 doses of 11.4 mg betamethasone (6 mg betamethasone sodium phosphate and 6 mg betamethasone acetate) or placebo equivalent; twice standard dose = 2 doses of 22.8 mg betamethasone (12 mg betamethasone sodium phosphate and 12 mg betamethasone acetate) or placebo equivalent

Diagnosis or treatment of a mental health disorder occurred in 96/229 (41.9%) of betamethasone-exposed participants and 84/195 (43.1%) of placebo-exposed participants (aRR 1.00, 95% CI 0.79, 1.26, *p* = 0.99). A similar proportion in each group had at least one inpatient admission for a mental health disorder (betamethasone group 11/223, 4.9% vs placebo 8/188, 4.3%) or were dispensed pharmaceuticals for mental health disorders (betamethasone group 72/229, 31.4% vs placebo 65/193, 33.7%) (Table 2). The prevalence of anxiety disorder and depression did not differ significantly between groups (anxiety, betamethasone group 68/229, 29.7% vs placebo 66/194, 34.0%; depression, betamethasone group 68/229, 29.7% vs placebo 66/194, 34.0%). Bipolar affective disorder and schizophrenia also did not differ significantly between treatment groups, although both were uncommon, with wide CIs around the estimate of effect (bipolar disorder aRR 0.34 (95% CI 0.08, 1.44, *p* = 0.13), schizophrenia aRR 1.52 (95% CI 0.13, 18.02, *p* = 0.74)).

**Table 2:** Mental health outcomes

Outcome	Betamethasone *n*/*N* (%)	Placebo *n*/*N* (%)	Unadjusted relative risk (95% CI)	Adjusted relative risk (95% CI)^a^	*P* value (adjusted)
Diagnosis or treatment of a mental health disorder	96/229 (41.9)	84/195 (43.1)	0.97 (0.78, 1.22)	1.00 (0.79, 1.26)	0.99
Depression	68/229 (29.7)	66/194 (34.0)	0.87 (0.66, 1.16)	0.91 (0.68, 1.23)	0.53
Bipolar affective disorder	3/229 (1.3)	7/195 (3.6)	0.37 (0.10, 1.40)	0.34 (0.08, 1.44)	0.13
Anxiety disorder	44/229 (19.2)	26/195 (13.3)	1.44 (0.92, 2.25)	1.51 (0.94, 2.42)	0.083
Schizophrenia	2/229 (0.9)	1/195 (0.5)	1.70 (0.15, 18.8)	1.52 (0.13, 18.02)^b^	0.74
Inpatient admission for a mental health disorder	11/223 (4.9)	8/188 (4.3)	1.16 (0.47, 2.83)	1.07 (0.41, 2.82)	0.88
Prescriptions of pharmaceuticals for mental health disorders	72/229 (31.4)	65/193 (33.7)	0.93 (0.71, 1.23)	0.95 (0.77, 1.28)	0.73

*CI* confidence interval

^a^Analyses adjusted for sex, gestational age at trial entry and for clustering

^b^The clustering term was removed from the analysis as the model failed to converge

At least one bone fracture occurred in similar numbers of betamethasone-exposed (143/226, 63.3%) and placebo-exposed (118/192, 61.5%) participants (aRR 1.01, 95% CI, 0.86, 1.19, *p* = 0.89), with no difference in the median number of fractures (Table 3) or at least one fracture at an osteoporotic site (39.6% [89/225] in the betamethasone-exposed and 31.8% [61/191] in the placebo-exposed participants, aRR 1.24, 95% CI 0.93, 1.66, *p* = 0.13). However, at least one fracture at an osteoporotic site that occurred at 18 years or older was more common in the betamethasone group (53/223, 23.8%) than in the placebo group (27/190, 14.2%) (aRR 1.57 95% CI 1.00, 2.48, *p* = 0.05). Post hoc subgroup analysis did not provide evidence of a dose–response effect for this outcome (Additional file 4, Table S1).

**Table 3:** General health outcomes

**Outcome**	**Betamethasone**	**Placebo**	**Unadjusted RR or MD (95% CI)**	**Adjusted RR or MD (95% CI)**^**a**^	***P*** **value** **(adjusted)**
Cancer diagnosis,*n*/*N* (%)	28/229 (12.2)	18/195 (9.2)	RR 1.32 (0.76, 2.32)	RR 1.40 (0.77, 2.55)	0.25
Allergic condition other than asthma (diagnosis of or prescription of treatments for allergic conditions), *n*/*N* (%)	127/229 (55.5)	108/193 (56.0)	RR 0.99 (0.84, 1.18)	RR 1.03 (0.86, 1.23)	0.76
Chronic kidney disease, *n*/*N* (%)	15/167 (9.0)	9/140 (6.4)	RR 1.40 (0.63, 3.10)	RR 1.36 (0.52, 3.54)	0.48
Self-reported oral health fair/poor, *n*/*N* (%)	79/223 (35.4)	56/192 (29.2)	RR 1.21 (0.91, 1.61)	RR 1.22 (0.92, 1.64)	0.15
Number of teeth removed for decay, median (5^th^, 95^th^ centile)	0 (0, 14)	0 (0, 12)	MD 0.6 (− 0.5, 1.8)	MD 0.6 (− 0.7, 1.8)	0.36
One or more teeth removed for decay, *n*/*N* (%)	99/214 (46.3)	83/185 (44.9)	RR 1.03 (0.83,1.28)	RR 1.00 (0.81, 1.26)	0.94
Total number of fractures,median (5^th^, 95^th^ centile)	1 (0, 4)	1 (0, 4)	MD 0.0 (− 0.3, 0.3)	MD 0.0 (− 0.3, 0.4)	0.85
Total number of fractures, *n*/*N* (%)			NA	NA	0.82
0	83/226 (36.7)	74/192 (38.5)			
1	61/226 (27.0)	59/192 (30.7)			
2	43/226 (19.0)	21/192 (10.9)			
3	21/226 (9.3)	17/192 (8.9)			
4	17/192 (8.9)	12/192 (6.3)			
≥ 5	11/226 (4.9)	9/192 (4.7)			
At least one fracture, *n*/*N* (%)	143/226 (63.3)	118/192 (61.5)	RR 1.03 (0.89, 1.20)	RR 1.01 (0.86, 1.19)	0.89
At least one fracture of osteoporotic sites, *n*/*N* (%)	89/225 (39.6)	61/192 (31.8)	RR 1.25 (0.96, 1.62)	RR 1.24 (0.93, 1.66)	0.13
At least one fracture of osteoporotic sites at age 18 years or older, *n*/*N* (%)	53/223 (23.8)	27/190 (14.2)	RR 1.67 (1.10, 2.55)	RR 1.57 (1.00, 2.48)	0.048
Functional difficulties, *n*/*N* (%)					
Difficulty seeing	6/223 (2.7)	3/191 (1.6)	RR 1.71 (0.43, 6.78)	RR 1.78 (0.40, 7.94)	0.42
Difficulty hearing	7/223 (3.1)	4/192 (2.1)	RR 1.51 (0.45, 5.09)	RR 1.56 (0.42, 5.83)	0.48
Difficulty walking	13/223 (5.8)	9/192 (4.7)	RR 1.24 (0.54, 2.85)	RR 1.32 (0.52, 3.35)	0.53
Difficulty remembering	6/223 (2.7)	8/192 (4.2)	RR 0.65 (0.23, 1.83)	RR 0.76 (0.24, 2.39)	0.62
Difficulty washing all over	5/223 (2.2)	3/192 (1.6)	RR 1.44 (0.35, 5.95)	RR 1.84 (0.40, 8.44)	0.41
Difficulty communicating	3/223 (1.4)	2/192 (1.0)	RR 1.29 (0.22, 7.69)	RR 1.39 (0.22, 8.98)	0.71
Any functional difficulties, *n*/*N* (%)	26/223 (11.7)	19/192 (9.9)	RR 1.18 (0.67, 2.06)	RR 1.29 (0.70, 2.37)	0.39
Physical activity, *n*/*N* (%)			NA	NA	0.14
Less than 30 min per week	44/222 (19.8)	38/192 (19.8)			
30 to 150 min per week	48/222 (21.6)	27/192 (14.1)			
At least 150 min per week	130/222 (58.6)	127/192 (66.2)			
At least 300 min per week	102/222 (46.0)	97/192 (50.5)	RR 0.91 (0.74, 1.11)	RR 0.87 (0.71, 1.07)	0.17

*CI* confidence intervals, *MD* mean difference, *NA* not applicable, *NE* not estimable, *RR* relative risk

^a^Analyses adjusted for sex, gestational age at trial entry and for clustering

Risk of a cancer diagnosis was similar between treatment groups (aRR 1.40, 95%Ci 0.77, 2.55, *p* = 0.25) (Table 3). A post hoc sensitivity analysis including participants who had died prior to study follow-up did not change the findings (Additional file 4, Table S2).

At least one self-reported functional difficulty occurred in 26/223 (11.7%) of those in the betamethasone group and 19/192 (9.9%) of the placebo group (aRR 1.29, 95% CI 0.70, 2.37, *p* = 0.39). The prevalence of specific functional difficulties ranged from 1.2% (5/415) for difficulty communicating to 5.3% (22/415) for difficulty walking and was similar in both groups (Table 3).

The proportion of participants with physical activity of < 30, 30–150 and > 150 min per week was similar between intervention groups (*p* = 0.14) as was the proportion with physical activity of at least 300 min per week (aRR 0.87, 95% CI 0.71, 1.07, *p* = 0.17) (Table 3).

Other general health outcomes were similar between groups, including allergic conditions other than asthma, chronic kidney disease and oral health outcomes (Table 3).

Tertiary qualification attainment was not significantly different between participants exposed to betamethasone 126/223 (56.5%) and placebo 113/193 (58.6%) (aRR 0.97, 95% CI 0.81, 1.16, *p* = 0.75). The proportion with neither secondary nor tertiary qualifications, employment status and the proportion with one or more criminal convictions were not different between intervention groups (Table 4).

**Table 4:** Educational and socioeconomic outcomes

Outcome	Betamethasone *n*/*N* (%)	Placebo *n*/*N* (%)	Unadjusted relative risk (95% CI)	Adjusted relative risk (95% CI)^a^	*P* value (adjusted)
Highest level of educational attainment			NA	NA	0.74
Tertiary	126/223 (56.5)	114/193(59.1)			
Secondary	65/223 (29.2)	51/193 (26.4)			
Neither tertiary nor secondary	32/223 (14.4)	28/193 (14.5)			
Proportion with highest level of education tertiary	126/223 (56.5)	114/193 (59.1)	0.96 (0.81, 1.13)	0.96 (0.81, 1.15)	0.66
No secondary or tertiary school qualification	32/223 (14.4)	28/193 (14.5)	0.99 (0.62, 1.58)	0.99 (0.59, 1.65)	0.97
Any criminal conviction	7/191 (3.7)	4/166 (2.4)	1.52 (0.45, 5.13)	1.28 (0.34, 4.79)	0.68
Employment status					0.94
Working in paid employment	187/220 (85.0)	158/189(83.6)	-	-	
Retired/homemaker/caregiver/full-time student	9/220 (4.1)	6/189 (3.2)	-	-	
Not in paid work/retired/ homemaker/caregiver/full-time student	24/220 (10.9)	25/189 (13.2)	-	-	
Proportion not in paid work, retired, full-time student or caregiver	24/220 (10.9)	25/189 (13.2)	0.82 (0.49,1.40)	0.87 (0.48,1.56)	0.61

*CI* confidence intervals, *NA* not applicable

^a^Analyses adjusted for sex, gestational age at trial entry and for clustering

## Discussion

The AST was the first and remains one of the largest randomised controlled trials of ANC for women at risk of preterm birth. In 424 adult survivors born to mothers who took part in the AST, we found no important differences at 50 years of age between those exposed to betamethasone or placebo for mental health, general health and social outcomes.

There has been particular concern about mental health outcomes after exposure to ANC. One observational cohort of very low birthweight infants reported a higher risk of depression at 26–30 years in those exposed to ANC but no differences in anxiety disorders, suicidal ideation or substance use disorders [13]. Another cohort study of 179 extremely low birthweight participants found a higher risk of clinically significant anxiety scores at 29–36 years in ANC-exposed participants compared to those not exposed [26]. Large population-based studies have also found a higher risk of behavioural and mental disorders in children exposed to antenatal corticosteroids compared with matched controls [14, 27, 28].

In contrast, we identified no significant effect of ANC exposure on the prevalence of mental health diagnoses, pharmaceuticals dispensing or inpatient admissions, although for anxiety disorders the findings are potentially consistent with an increase in risk with ANC, but with wide CIs precluding statistical significance, possibly due to limited sample size. For mental health outcomes, we had access only to self-reported questionnaires, hospital admissions and pharmaceutical dispensing data. Hospital admissions data will only identify mental health diagnoses referred to in the clinical record during an admission, meaning undiagnosed or community-managed mental illnesses or those not mentioned in clinical records will be omitted. Pharmaceutical dispensing data is non-specific as many pharmaceuticals are used for the treatment of multiple different mental health diagnoses. Nevertheless, our data suggest that ANC exposure does not increase the overall risk of mental health disorders to 50 years of age.

These findings are consistent with those of other reports of randomised trials, which differ from those of the observational studies. Psychological testing at 30 years of age in the AST cohort did not identify any differences between intervention groups in cognitive functioning, working memory, attention or psychiatric morbidity [29]. A 20-year follow-up of 81 participants of another randomised trial of ANC also showed no differences between intervention groups for cognitive functioning or psychiatric symptoms [30]. Similarly, a randomised trial of ANC for elective caesarean section at term showed no difference in behavioural difficulties in 402 children followed up at 8 to 15 years of age [31].

In contrast, animal studies of long-term outcomes of ANC exposure have reported negative neurobiological and behavioural consequences in the offspring, including behaviour analogous to anxiety, changes in motor behaviour in rodents and changes in social behaviour in both rodents and non-human primates [32]. These have been ascribed to changes in glucocorticoid and mineralocorticoid receptor concentrations in the hypothalamus, hippocampus and amygdala, and reduced neuron concentrations in the serotonergic and dopaminergic systems [32]. The discrepancy between these marked effects in pre-clinical studies and the findings of no detectable effects in randomised human studies may be reflected in part by differences in the timing of the dose during gestation and use of ANC doses greatly in excess of those used in clinical practice [9–11, 33–35].

We also assessed a range of general health outcomes. There were no differences between treatment groups in mean number of fractures, the proportion with at least one fracture or the proportion with at least one fracture at an osteoporotic site. However, the betamethasone-exposed group were more likely to have had a fracture at an osteoporotic site in adulthood. There was no evidence of a relationship between betamethasone dose and the proportion with a fracture at an osteoporotic site in adulthood. This fracture definition was chosen as it excluded sites such as skull or finger fractures that are unlikely to be related to lower bone density or quality and excluded childhood fractures for which aetiology may differ from adult fractures [24]. Observational data have described a relationship between early life growth and nutrition, later bone mineral content (BMC) and later fracture risk [36]. The relationship between birthweight and later BMC is most well established, with lower birthweight associated with lower BMC in later life [37]. Animal studies have indicated changes in skeletal growth after ANC [35, 38–40], and human studies have indicated changes in bone turnover in the neonatal period [41, 42]. However, in a subset of 174 AST participants assessed with dual-energy X-ray absorptiometry at 30 years, when bone mass is likely close to its peak, there were no differences between intervention groups in bone mass or areal bone density [43]. Follow-up of a clinical trial of repeat doses of ANC compared with a single course at 6–8 years also did not find differences in bone mass between groups [44]. The lack of a dose–response effect of betamethasone on fracture risk in this study, no difference in peak bone mass at 30 years and no difference in total fractures in this study, along with the large number of comparisons made, makes it likely that our finding of increased osteoporotic site fractures in adulthood represents a type 1 error.

Evidence from a meta-analysis of three randomised trials suggests that antenatal corticosteroids may reduce childhood developmental delay, including items likely to contribute to later functional limitations such as cerebral palsy, blindness and deafness, but data on participant experience of functional limitations in later life are sparse [3]. A recent follow-up of a clinical trial of ANC for women at risk of late-preterm birth (34–37 weeks’ gestation) reported no difference in adverse neurodevelopmental outcomes between groups at 7 years of age [45]. Again, these findings contrast with those from observational studies that have indicated a greater risk of neurodevelopmental disorders in children exposed to ANC, but the confounding effects in observational studies of the indication for corticosteroid exposure are difficult to resolve [14, 27, 46, 47]. In this study, individual functional limitations were uncommon (1.2 to 5.3% prevalence), although 11% of participants had at least one functional limitation (45/415), with no differences between intervention groups. Since children and adolescents born moderate (32–34 weeks’ gestation) or late (34–37 weeks’ gestation) preterm, the majority of preterm births in the present cohort, have a higher risk of motor, cognitive and sensory impairment than those born at term [48], and given the age of the cohort, these rates are consistent with those found in New Zealand census data in the population aged over 5 years old, ranging from 1.0% (difficulty communicating) to 3.1% (difficulty walking or climbing steps) [49].

In observational studies, preterm birth is associated with worse renal function in adolescence and early adulthood, and animal studies have suggested a deleterious impact of ANC exposure on offspring nephron number and blood pressure in rodents and sheep [50–52]. However, an observational study of 162 adolescents with very low birthweight did not find a difference in renal function between ANC-exposed and non-exposed participants, except in those participants with elevated waist-to-hip ratio [53]. We did not find a difference in chronic kidney disease between treatment groups although the overall prevalence (24/307, 7.8%) was greater than population estimates for 50–59-year-olds (5.4%) [54].

All oral health outcomes assessed did not differ between treatment groups. Poor oral health is strongly associated with low socioeconomic status and has a considerable impact on quality of life, along with direct and indirect economic costs [55, 56]. To our knowledge oral health outcomes have not been studied previously in long-term follow-up of ANC exposure.

We found no difference in allergic conditions other than asthma between treatment groups, similar to the findings of a randomised, placebo-controlled clinical trial of antenatal betamethasone for elective caesarean at term, which followed up participants at 8 to 15 years old [31].

Educational and employment outcomes also did not differ between intervention groups. This is consistent with no difference in employment and educational outcomes reported in the earlier 30-year follow-up of this trial, and another randomised trial of ANC at 20 years [7, 30]. However, a randomised trial of ANC for elective caesarean birth at term reported a smaller proportion of students at 8–15 years in the top quarter of academic ability as assessed by the school in those exposed to ANC [31]. Previous follow-up of the AST participants also found no effect on IQ at 30 years in those exposed to ANC, as did a recent observational cohort of 250 adults aged 26–30 years born at very low birthweight [13, 29]. Similarly, there was no difference in the proportion with criminal convictions between those treated with ANC and those untreated in our study, or the observational cohort study at 26–30 years [13]. Together, these findings suggest that exposure to ANC does not have clinically important effects on these aspects of the ability to function in society in adulthood.

## Strengths and limitations

A key strength of this study is that it is the longest reported follow-up study of outcomes from a randomised clinical trial of antenatal corticosteroids, reducing the risk of bias from confounding. This is particularly important because indication for treatment and healthcare disparities are major and unquantifiable confounders in non-randomised, observational studies of the long-term effects of ANC.

Another strength is the diverse range of adult health and social outcomes, allowing us to explore multiple different pathways through which ANC could impact on later quality of life and function. However, assessing a large number of outcomes does increase the likelihood of type 1 error.

The use of both self-reported questionnaires and administrative data is also a strength of this study. Using administrative datasets to augment self-report reduces recall bias. For example, data on fractures from NMDS and ACC data allowed the inclusion of fractures that participants did not recall. However, important limitations of using administrative data for outcome ascertainment include the potential for misclassification of outcomes through miscoding of diagnoses or by defining outcomes based on treatments that are used for more than one condition. Administrative databases are also dependent on health care access, meaning outcomes defined by a treatment or diagnostic test assume similar access to health care across participants and may be biased if this is not the case.

An important potential limitation is the low follow-up rate (46% of those presumed to be alive), which increases the risk of attrition bias. However, this would only affect the reliability of our findings if loss to follow-up was differential between randomised groups. This is unlikely, since characteristics were similar between those followed up and those lost to follow-up, except for the expected differences in the group of deceased participants due to the higher early mortality for those born at earlier gestational ages.

The proportion of female offspring in the placebo group was higher than in the betamethasone group, likely related to the increased mortality of male preterm neonates, particularly prominent in those without antenatal corticosteroid treatment. This sex imbalance between treatment groups may have different impacts depending on the outcome measured. Mental health diagnoses differ by sex, as does the risk of fracture, criminal convictions and potential outcomes related to education and health behaviours. However, we included sex as a covariate in adjusted analyses, thus mitigating the effect of sex imbalance. In addition, most infants were born moderate to late preterm, meaning these findings are not necessarily applicable to those born at earlier gestational ages who generally experience greater morbidity. Nevertheless, those born moderate and late preterm comprise the majority of preterm births and are at greater risk of both neonatal and later-life morbidity than those born at term [57–60].

## Conclusions

We identified no clinically important effects on mental health, general health and social outcomes to 50 years of age after exposure to ANC. The neonatal benefits for mortality and morbidity and possible childhood neurodevelopmental benefits of antenatal corticosteroids administered for preterm birth do not appear to be offset by negative adult consequences.

## Data Availability

De-identified participant data will be available to researchers who provide a methodologically sound proposal with appropriate ethical approval, where necessary, and following approval of the proposal by the Data Access Committee at the Liggins Institute. Data requestors will be required to sign a data access agreement before data are released. Request for access to data can be made to the Maternal and Perinatal Research Hub at the Liggins Institute, University of Auckland (researchhub@auckland.ac.nz).

## Abbreviations

ACC: Accident Compensation Corporation
aMD: Adjusted mean difference
ANC: Antenatal corticosteroid
AST: Auckland Steroid Trial
aRR: Adjusted relative risk
CI: Confidence interval
IPAQ: International Physical Activity Questionnaire
GLM: Generalised linear modelling
MD: Mean difference
MORT: Mortality dataset
NMDS: National Minimum Dataset
NNPaC: National Non-admitted Patients Collection
NZCR: New Zealand Cancer Registry
NZQA: New Zealand Qualifications Authority
PHARM: Pharmaceutical dataset
RR: Relative risk
SD: Standard deviation
WGSS: Washington Group Short Set on Functioning
